# Suppressing male spermatogenesis-associated protein 5-like gene expression reduces vitellogenin gene expression and fecundity in *Nilaparvata lugens* Stål

**DOI:** 10.1038/srep28111

**Published:** 2016-06-16

**Authors:** Lin-Quan Ge, Ting Xia, Bo Huang, Qi-Sheng Song, Hong-Wei Zhang, David Stanley, Guo-Qing Yang, Jin-Cai Wu

**Affiliations:** 1 School of Plant Protection Yangzhou University, Yangzhou 225009, P.R. China; 2 Division of Plant Sciences, University of Missouri, 1-31 Agriculture Building, Columbia, MO 65211, USA; 3 USDA/Agricultural Research Service, Biological Control of Insects Research Laboratory, Columbia, Missouri, USA

## Abstract

In our previous study with the brown planthopper (BPH), *Nilaparvata lugens*, triazophos (tzp) treatments led to substantial up-regulation of a male spermatogenesis-associated protein 5-like gene (*NlSPATA5*) compared to untreated controls. Mating with tzp-treated males significantly increased fecundity (as numbers of eggs laid), relative to females mated with untreated males. Because SPATA5 acts in mammalian sperm development and is expressed in testes, we posed the hypothesis that *Nl*SPATA5 occurs in BPH seminal fluid and it operates in fecundity via mating. We tested the hypothesis by investigating the influence of suppressing *NlSPATA5* expression in BPH males on fecundity. Reduced expression of *NlSPATA5* led to decreased male accessory gland protein content and reproductive system development compared to controls. These changes in males led to prolonged pre-oviposition periods and decreased fecundity in females. For both genders, we recorded no difference in the body weight, oviposition periods, and longevity compared to controls. *NlSPATA5* suppression in males also led to decreased fat body and ovarian protein content, yeast-like symbionts abundance and ovarian development as well as vitellogenin gene expression in their mating partners. We infer that increased *Nl*SPATA5 expression may be one molecular mechanism of tzp-driven reproduction and population increases in BPH.

The brown planthopper (BPH), *Nilaparvata lugens* (Stål) is a serious pest of rice crops in Asia and Australia^1^ and a classic example of an insecticide-induced resurgent pest^2^. Previous studies have demonstrated that insecticide treatments enhanced BPH male accessory gland (MAG) protein content^3^. Compared to crossing with untreated males, females mating with insecticide-treated males led to increased fecundity, registered as numbers of eggs laid^3^. The effects of insecticides on BPH reproduction appear to operate through a spermatogenesis-associated protein 5-like (*NlSPATA5*) gene because it was up-regulated after triazophos (tzp)-treatment. However, a direct link between *NlSPATA5* up-regulation and female reproduction has not been investigated^4^. Nonetheless, because of its broad importance in animals, it has become a BPH gene of interest.

In insects, MAG proteins are essential components of seminal fluids that act in influencing post-mating changes in female behavior, such as reduced sexual receptivity^5^, increased oviposition^6^ and increased sexual refractory periods^7^. MAG proteins act in sperm transport and storage, and because they also include antimicrobial peptides, they provide prophylactic protection of the female reproductive tract^8^. Insect mating systems are evolved and tremendously varied traits. For a single example, MAG proteins do not induce refractoriness in all species^9^. MAG proteins also influence post-mating female physiology, stimulating oogenesis, ovulation and oviposition. The idea that males can influence their sexual partners via constituents of their seminal fluids has helped to understand the mating systems of many animal species and may have practical relevance for insect pest management technologies^10^.

Spermatogenesis-associated proteins (SPATAs) were discovered in research designed to identify new spermatogenesis-associated genes. This work with a human testis cDNA library yielded a protein-encoding gene first called PD1 and later renamed *spata2*, later shown to operate in spermatogenesis and pancreatic islets^11^. Several *spata*s, and RNA splicing variants, have been identified, most of which act in diverse areas of testicular biology. For one example, SPATA6 is thought to act in testicular germ cell tumors in a human embryonic carcinoma cell line^12^. SPATA proteins also occur in insects, identified among a range of genes associated with spermatogenesis in *Drosophila* as well as human genomes^13^. In a similar vein, treating BPH with the organophosphate, tzp, led to increased expression of several proteins, including a SPATA5-like protein^4^. This finding led to hypotheses about the possible biological significance of SPATA5 in BPH.

We investigated the possibility that a single male gene product, *Nl*SPATA5, influences parameters of female reproductive biology. If this idea were strongly supported, the gene encoding *Nl*SPATA5 could potentially serve as a target for RNAi based control of insect pests in transgenic plants. We posed this concept as the hypothesis that *Nl*SPATA5 occurs in BPH seminal fluid and it operates in fecundity via mating. If this idea were strongly supported in the future research, suppressing *NlSPATA5* expression will influence the post-mating behavior and physiology of female partners, which would reduce BPH fitness in agroecosystems. Here we report on the outcomes of experiments designed to test our hypothesis.

## Results

### Expression analysis of *NlSPATA5*

**Figure 1:**
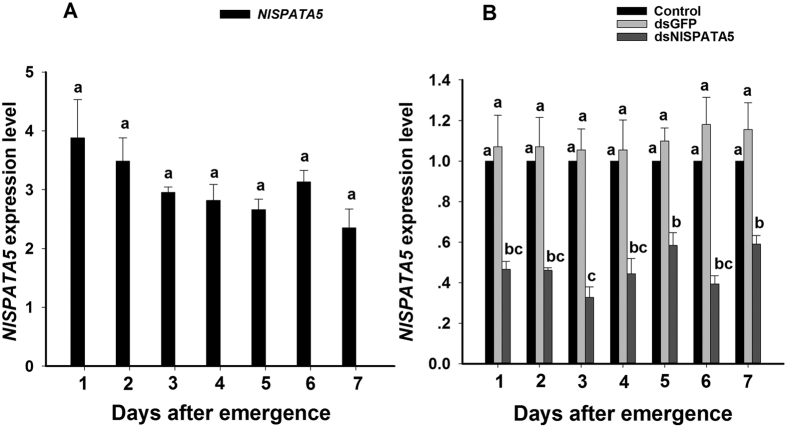
Expression of *NlSPATA5* (Panel **A**) and the influence of dietary dsNlSPATA5 on expression of the cognate gene (Panel **B**) during the first seven days following adult male emergence. *NlSPATA5* expression value of untreated controls males was converted to 1. The histogram bars show mean relative gene expression (n = three independent biological replicates) and the error bars represent one standard deviation (t-test, *P* < 0.05). Gene expression was normalized to the β-actin reference gene.

Although it appears that *NlSPATA5* expression declined with age in adult males, statistical analysis confirmed the abundances of mRNA encoding *Nl*SPATA5 remained at the same level from days 1 to 7 following adult emergence (*F* = 2.3, df = 6, 20, *P* = 0.10; Fig. 1A). We used cDNA prepared from unmated females as negative controls in which we detected no *NlSPATA5* transcripts.

Analysis of abundances of mRNA encoding NlSPATA5 in untreated controls, dsGPF-treated controls and NlSPATA5-treated males confirmed that dietary dsNlSPATA5 treatments significantly down-regulated *NlSPATA5* expression in adult males for the first seven days of adulthood (*F* = 374.9, df = 2, 42, *P* = 0.0001) (Fig. 1B). To evaluate the effect of dietary dsRNA on *NlSPATA5* mRNA abundances, the expression value of untreated controls was converted to 1. Dietary dsNlSPATA5 treatments led to reduced *NlSPATA5* expression in males, by about 41~67% compared to untreated controls, and by approximately 46~68% compared to dsGFP control over the seven day period. The average of *NlSPATA5* expression was not significantly influenced by interaction effect between DPE and dsRNA treatments (*F* = 1.6, df = 12, 42, *P* = 0.13).

### The influence of dietary dsNlSPATA5

**Figure 2:**
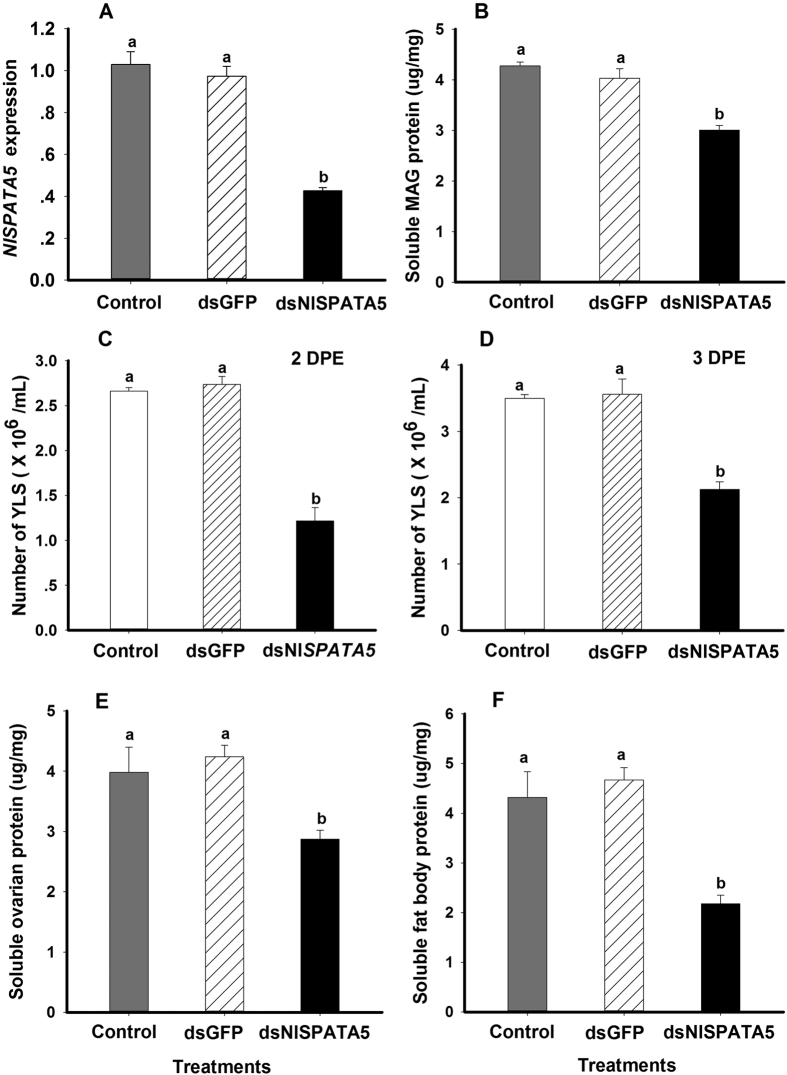
Influence of dietary ds*NlSPATA5*. Panel (**A**) the histogram bars show mean relative gene expression ± S.E (n = three independent biological replicates) at 2 DPE. Panel (**B**) the histogram bars show mean MAG protein content (mg/g tissue, ± S.E, n = 4 independent biological replicates) at 2 DPE. Panels (**C,D**) the histogram bars indicate mean numbers of YLS at 2 DPE and 3 DPE. Panel (**E**) Mean soluble ovarian protein content (mg/g) at 2 DPE. Panel (**F**) Mean soluble fat body protein content (mg/g) at 2 DPE. Histogram bars annotated with the same letter are not significantly different (t-test, *p* < 0.05).

Dietary dsNlSPATA5 treatments led to significantly reduced abundances of mRNA encoding *NlSPATA5* in males at day 2 post-emergence (2 DPE) as just shown (Fig. 2A) (*F* = 54.4, df = 2, 8, *P* = 0.0001), down by about 56% compared to untreated controls and by approximately 58% compared to dsGFP controls (Fig. 2A). Dietary dsNlSPATA5 treatments led to significantly decreased soluble MAGs protein contents at 2 DPE (Fig. 2B) (*F* = 106.4, df = 2, 8, *P* = 0.0001), down by circa 30% (from 4.27 μg/mg to 3.01 μg/mg), compared to controls and by 25% (from 4.03 μg/mg to 3.01 μg/mg), compared to dsGFP-treated controls, again at 2 DPE (Fig. 2B).

Mating with males exposed to similar *NlSPATA5-*suppressing treatments led to significantly decreased YPS abundance of fat bodies in adult females at 2 and 3 DPE (Fig. 2C,D) (*F* = 342.0, df = 2, 14, *P* = 0.0001 for 2DPE; *F* = 143.8, df = 2, 4, *P* = 0.0001 for 3 DPE), down by 54% (from 2.66 × 10^6^/mL to 1.22 × 10^6^/mL) compared to untreated controls and by approximately 55% (from 2.73 × 10^6^/mL to 1.22 × 10^6^/mL) relative to dsGFP-treated controls at 2 DPE. By 3 DPE, YPLs were down by 39% from 3.50 × 10^6^/mL to 2.13 × 10^6^/mL) compared to mating with untreated controls and by approximately 40% (from 3.56 × 10^6^/mL to 2.13 × 10^6^/mL) compared to mating with dsGFP-treated controls.

Dietary dsNlSPATA5 treatments in males led to significantly decreased soluble ovarian protein content and soluble fat bodies protein content in their mating partners at 2 DPE (Fig. 2E,F) (F = 6.82, df = 2, 11, *P* = 0.016 for ovary; *F* = 60.5, df = 2, 11, *P* = 0.0001 for fat body). Mating with males treated with dietary ds*NlSPATA5* led to reduced soluble ovarian protein content, down significantly by 27% (from 3.98 μg/mg to 2.87 μg/mg) compared to untreated controls and by approximately 32% (from 4.24 μg/mg to 2.87 μg/mg) relative to dsGFP-treated male controls (Fig. 2E) at 2 DPE. We recorded similar reductions in soluble fat body protein content, down by about 48% (from 4.32 μg/mg to 2.17 μg/mg) compared to untreated controls and by approximately 53% (from 4.67 μg/mg to 2.17 μg/mg) compared to dsGFP-treated controls (Fig. 2F) at 2 DPE.

### Dietary dsNlSPATA5 led to malformed reproductive systems

**Figure 3:**
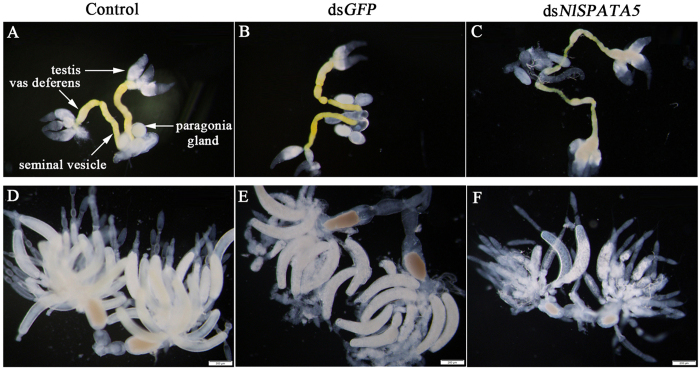
Dietary ds*NlSPATA5* led to malformed reproductive systems at 2 DPE. The third instar nymphs were treated with dietary ds*NlSPATA5*. Panels (**A–C**) Reproductive tracts were isolated from males and photographed using an OLYMPUS SZX16 microscope. We note the reduced sizes of the vas deferens and seminal vesicle. Panels (**D–F**) Reproductive tracts were isolated from females and photographed using an OLYMPUS SZX16 microscope. We note the reduced sizes of ovaries.

Compared to untreated males (Fig. 3A) and dsGFP-treated males (Fig. 3B), dietary dsNlSPATA5 led to significant malformation of vas deferens and seminal vesicle in males at 2 DPE (Fig. 3C), but no externally visible effect on testes and accessory glands was observed (Fig. 3C).

The external morphology of ovaries prepared from females after mating with experimental males, was also influenced by dietary dsRNA construct. The ovarioles within ovaries of females that mated with untreated males (Fig. 3D) or with dsGFP-treated males, (Fig. 3E), contained one or two fully developed banana-shaped oocytes at 2 DPE. However, mating with males exposed to dietary dsNlSPATA5 treatments resulted in undeveloped ovaries and severely inhibited oocyte growth in the ovaries (Fig. 3F). No fully developed oocytes were observed at 2 DPE in the dsNlSPATA5 group (Fig. 3F).

### dsNlSPATA5 did not influence adult body weight or longevity

**Figure 4:**
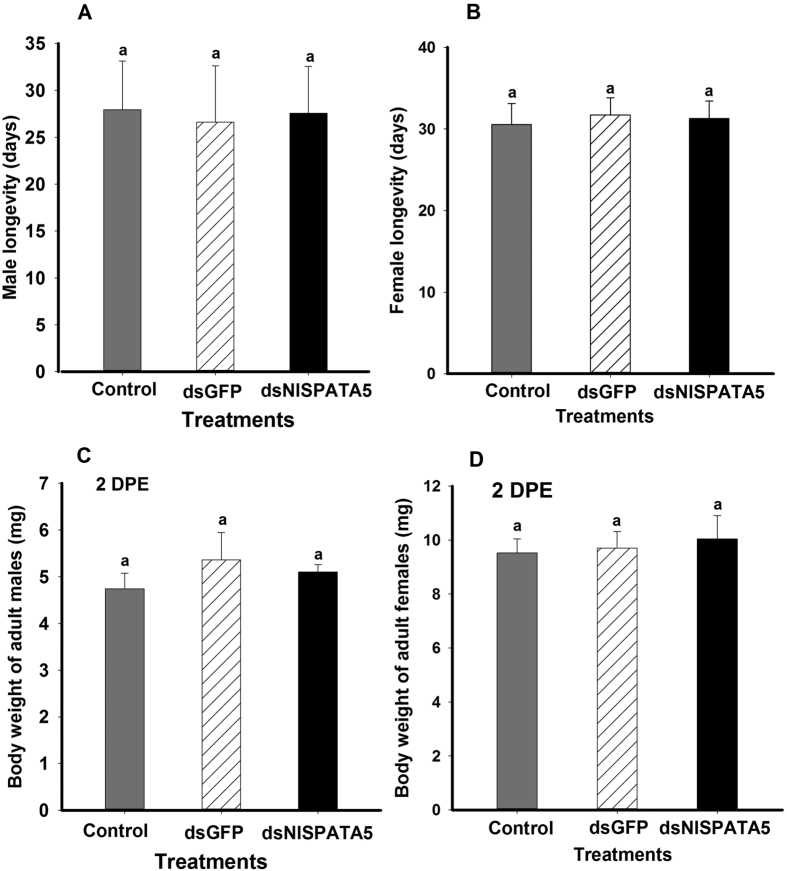
The influence of dietary ds*NlSPATA5* on male longevity (Panel **A**) and female longevity (Panel **B**) at day 2 pe. Panel (**A**) and Panel (**B**) histogram bars show the mean longevity ± S.E. (n = five independent biological replicates (5 males or 5 females/replicate). The influence of dietary ds*NlSPATA5* on male body weight (Panel **C**) and female body weight (Panel **D**): Histogram bars show mean fresh body weight (n = 18 independent biological replicates). Histogram bars annotated with the same letters are not significantly different at *p* < 0.05.

Dietary dsNlSPATA5 treatments did not influence adult longevity (Fig. 4A,B) (*F* = 0.29, df = 2, 53, *P* = 0.07 for males; *F* = 0.21, df = 2,53, *P* = 0.81 for females) and body weight (Fig. 4C,D) (*F* = 3.0, df =2,14, *P* = 0.09 for males; *F* = 0.75, df = 2,14, *P* = 0.49 for females).

### Influence of dietary dsNlSPATA5 on reproduction parameters

**Figure 5:**
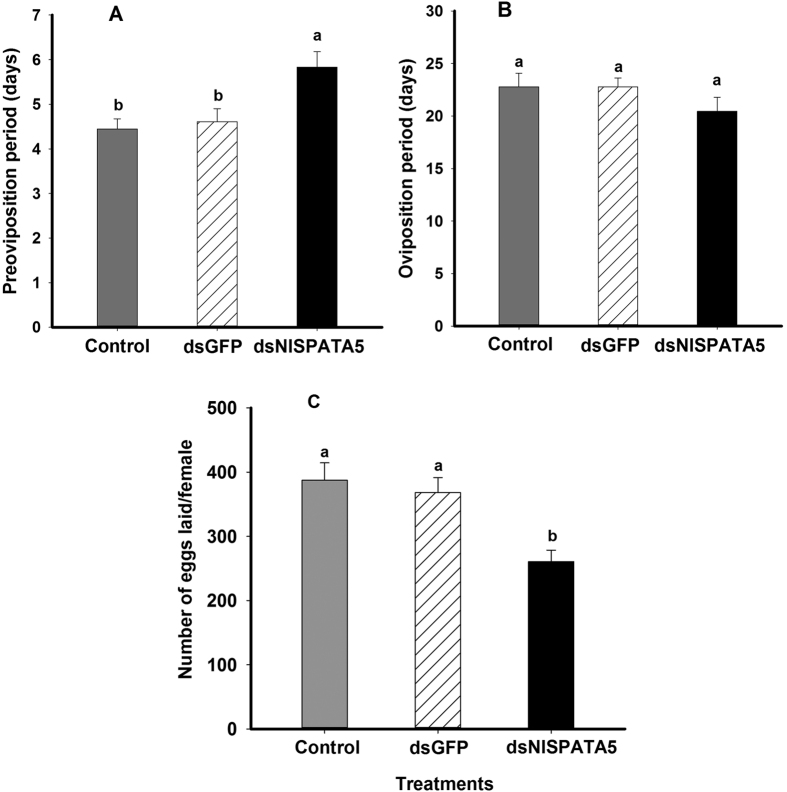
Influence of dietary ds*NlSPATA5* on reproduction parameters. Panel (**A**) The dietary ds*NlSPATA5* treatments led to increased pre-oviposition periods. Panel (**B**) The ds*NlSPATA5* treatments did not influence the oviposition period. Panel (**C**) The ds*NlSPATA5* treatments led to reduced fecundity. Histogram bars represent mean number of days (Panel **A,B**) or numbers of eggs (Panel **C**) +SEM. Histogram bars annotated with the same letter are not significantly different at *p* < 0.05.

The pre-oviposition period is the time, in days, from adult emergence to the onset of egg-laying. Mating with males exposed to dietary dsNlSPATA5 treatments prolonged the pre-oviposition period of females (*F* = 6.7, df = 2, 53, *P* = 0.003), increasing by 1.4 day (from about 4.4 to 5.8 days), compared to untreated controls (delayed by circa 31%) and dsGFP control (delayed by about 26%) (Fig. 5A).Dietary dsNlSPATA5 treatments did not influence the oviposition period (Fig. 5B) compared to untreated and dsGFP controls (*F* = 1.3, df = 2, 53, *P* = 0.29).

The dsNlSPATA5 treatments significantly decreased the number of laid eggs adult females (*F* = 9.4, df = 2, 53, *P* = 0.0003), down by 32% (from 387.4 eggs/female to 260.1 eggs/female) compared to untreated females and by approximately 29% compared to dsGFP females (from 368.2 eggs/female to 260.1 eggs/female) (Fig. 5C).

### Dietary dsNISPATA5 led to reduced *Nlvg* mRNA expression

**Figure 6:**
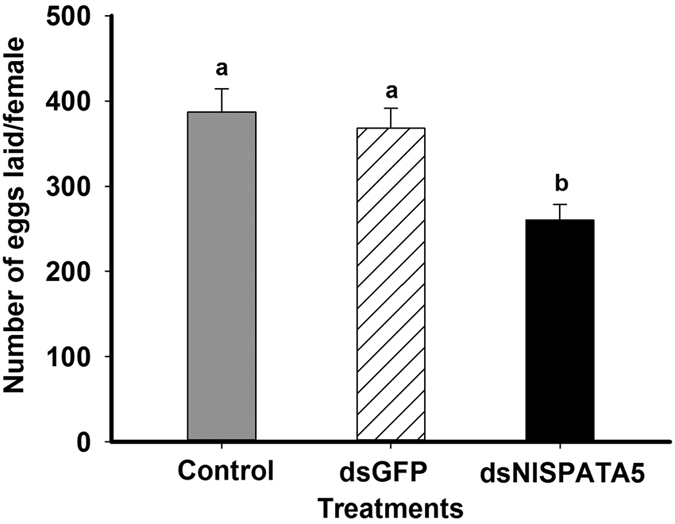
Mating with males exposed to dietary ds*Nlst6* led to decreased *Nlvg* mRNA expression at 2 DPE. Values were normalized relative to the reference gene, β-actin. Histogram bars represent the mean values ± SE (n = three replicates). Histogram bars annotated with the same letter are not significantly different (t-test, *p* < 0.05).

We examined the expression level of *Nlvg* mRNA in adults mating with dsNlSPATA5-treated and control males. Mating with males exposed to dietary dsNlSPATA5 treatments led to significantly down-regulated *Nlvg* expression at 2 DPE (*F* = 13.8, df = 2, 8, *P* = 0.006), down by 34% compared females that mated with untreated males and by approximately 30% compared to females that mating with dsGFP-treated males at 2 DPE (Fig. 6).

## Discussion

The data reported in this paper strongly support our hypothesis that suppressing *NlSPATA5* expression in males will influence the post-mating behavior and physiology of female partners, which would reduce BPH fitness in agroecosystems. Our data show that 1/*NlSPATA5* is expressed at substantial levels for at least the first seven days following male adult emergence; 2/dietary dsNlSPATA5 treatments led to reduced *NlSPATA5* expression, reproductive system development and Acps protein contents in males; 3/similar treatments with experimental males led to reduced numbers of YLS, ovarian development and soluble ovarian and soluble fat body protein contents in their untreated female partners; 4/dietary dsNlSPATA5 treatments in males altered female reproductive biology, including increased length of preoviposition periods and reduced *Nlvg* expression and egg laying. These treatments did not influence adult longevity, body weights or the oviposition periods. Taken together, these data indicate that suppressing expression of *NlSPATA5* in males strongly altered some, but not all, aspects of female reproductive biology. The significance of this finding lies in a direct demonstration of the influence of a single male-derived protein on female mating partners.

SPATA5 is a member of the ATPase associated with diverse activities protein super-family (AAA-protein super-family). There are five major groups of AAA-proteins that evolved in every form of life, including viruses. They act in such varied functions as organelle membranes, DNA replication and repair, protein degradation, and cilia and flagellar movement^14^. SPATA5, also known as spermatogenesis associated factor (SPAF), and ATPase family protein 2 homolog, was first discovered in mouse testes, where it is expressed in the early stages of spermatogenesis. It may also act in functional mitochondrial transformations during spermatogenesis^15^. The biological significance of SPATA5 goes far beyond spermatogenesis, however, because mutations in human SPATA5 lead to susceptibility to the hair loss disorder, alopecia areata^16^ and to microcephaly, intellectual disability and other serious disorders^17^, as well as in transformation to cancer cells^15^. Although SPATA5 is understudied in insect systems, the gene may be expressed in several insect tissues, including reproductive tissues. Our findings indicate that sexually-transferred NlSPATA5 is necessary to achieve full reproductive potential in the BPH mating system.

Work on individual genes and on global protein expression in insecticide- and jingGANGmycin-treated BPH^3^,^4^,^6^,^18^ demonstrates the enhancing influence of agricultural chemicals on BHP reproduction and population changes. The influence of these chemicals operates through multiple systems, including carbohydrate transport^19^, protein biosynthesis^18^, developmental hormones^18^ and lipid biosynthesis^20^. Treating males with either of the insecticides, the organophosphate tzp or the pyrethroid, deltamethrin, led to increased protein contents in treated males and in untreated females after mating with treated males^3^. These treatments also led to increased fecundity in untreated females after mating with treated males. This work establishes the idea that male BPHs transfer fecundity-improving proteins to their sexual partners.

*NlSPATA5* is expressed throughout the first seven days of male adulthood, indicating the protein is available for sexual transfer to females during each mating. Dietary dsNlSPATA5 led to reduced expression of the cognate gene and inhibition of vas deferens and seminal vesicle development, which in turn led to reduced Acps proteins in males. Suppressing *NlSPATA5* expression in males strongly influenced females, with reduced ovarian and fat body protein contents and inhibition of oocyte growth in the ovarioles. We emphasize these effects do not follow from toxic mechanisms because dsNlSPATA5 treatments did not influence body weight or longevity of males or their female partners. Proteins, of course, are critical to achieve full reproductive potentials. We registered the reduced ovarian and fat body protein contents in terms of fecundity, showing substantial reductions in numbers of eggs deposited by females whose mating partners had been treated with dietary dsNlSPATA5. Understanding that cells produce thousands of proteins raises questions about which proteins may be lacking in BPH partners that mated with ds*NlSPATA5*-treated males. For a first look at how the ds*NlSPATA5* treatments in males influence protein contents in females, we considered the yolk protein, vitellin (Vt) because it is a specific protein within ovarian follicles and acts exclusively in egg development. Vitellogenin (Vg) is generally produced in fat body and becomes Vt in the process of receptor-mediated uptake from hemolymph circulation into eggs. The relative expression of *Nlvg* encoding Vg was reduced in females after mating with dsNlSPATA5-treated males. We infer that *NlSPATA5* encodes a male-specific AAA-family protein that is necessary in females for biosynthesis of Vg and possibly many other proteins used to achieve the full reproductive potential of BPH.

Our findings show that dietary dsNlSPATA5 treatments reduced YLS abundance in females. A substantial relationship between YLS and amino acid requirements in BPH has been documented^21^,^22^. Meeting essential amino acid requirements was associated with the abundance of YLS^22^. Artificial reduction of the symbiont abundance led to delayed growth and decreased survival, adult emergence, body weight, and fecundity^22^. Symbiotic bacteria in pea aphids also significantly influenced amino acid metabolism^23^. Generally, the YLS provide essential amino acids and possibly proteins required for host embryo formation and post-embryonic development^24^. The symbionts are essential to meet nutritional amino acid needs for production of a full complement of proteins. We infer dietary dsNlSPATA5 treatment in males leads to reduced transfer of accessory gland proteins (including nutrition substances) to females via mating.

We help appreciate the biological significance of the sexual transfer of *Nl*SPATA5 by placing this finding in the context of insect mating systems, many of which include mechanisms that enhance female fecundity and ensure paternity^25^, ranging from nuptial gifts to mate guarding to substances transferred to females within seminal fluids. For a single example, the Australian field cricket, *Teleogryllus commodus*, mating system involves male calling, which attracts receptive females to the males’ mating arenas. Mating occurs via a spermatophore, which delivers seminal fluids into the spermatheca. The seminal fluids include sperm, a specific protein, prostaglandin (PG) synthase, and the lipid substrate for PG synthesis, arachidonic acid (AA). The AA is converted into PG within the spermatheca, which is transported in hemolymph to the terminal abdominal ganglion, where it releases a sequential behavioral program that ends in deposition of hundreds of fertilized eggs over the next several hours. Males ensure paternity by guarding their partners until the egg-laying program is completed^26^. While *Nl*SPATA5 does not appear to influence female sexual behaviors, it exerts profound molecular and biochemical effects on production of Vg and likely other proteins and these effects can be directly registered at the levels of organismal reproduction and BPH population dynamics.

## Materials and Methods

### Rice variety and culture

Rice (*Oryza sativa* L.) variety Ninjing 4 (japonica rice, commonly grown in Jiangu province) was used in all experiments. Seeds were sown outdoors in cement tanks (height 60 cm, width 100 cm, and length 200 cm) containing standard rice-growing soil. When seedlings reached the six-leaf stage, they were transplanted into 16 cm diameter plastic pots containing four hills per pot, three plants per hill and used for experiments at the tillering stage.

### Insect culture

BPHs were obtained from a laboratory population maintained in a greenhouse under our standard conditions (26 ± 2 °C, with 70–80% humidity and a 16L:8D photoperiod) at Yangzhou University. The insect colony was originally obtained from the China National Rice Research Institute (Hangzhou, China). Before the experiments started, the colony was allowed to reproduce for two generations in cement tanks (60 × 100 × 200 cm) under natural condition in Yangzhou.

### dsRNA

We designed gene-specific dsNlSPATA5 primers and amplified a 407-bp (422–828bp) *NlSPATA5* cDNA fragment using forward and reverse primers containing the T7 primer sequence at the 5′ ends (Table 1). The amplification program was 35 cycles of 95 °C for 40 s, 57 °C for 40s and 72 °C for 1 min, with a final extension step of 72 °C for 10 min. The sequence was verified by sequencing (Invitrogen, Shanghai, China). We used the GFP gene (ACY56286; provided by Zhang Chuan-xi, Institute of Insect Sciences, Zhejiang University) as control dsRNA and amplified a 688 bp fragment using primers listed in Table 1. For *NlSPATA5* and the control GFP gene, we used the T7 RiboMAX^TM^ Express RNAi System (Promega, Sunnyvale, CA) for dsRNA synthesis, following the Promega instructions. We generated sense and antisense dsRNAs in separate 20 μL reaction volumes. The dsRNAs were annealed by mixing and incubating at 70 °C for 10 min, and then cooling to room temperature over 20 min. 2 μL RNase A solution (4mg/ml) and 2 μL RNase-free DNase (1 u/μL) were added to the reaction tube and incubated in a 37 °C water bath for 30 min. The dsRNA was precipitated by adding 110 μL 95% ethanol and 4.4 μL 3 M sodium acetate (pH 5.2), then washed with 0.5 mL 70% ethanol and dried at room temperature. The dried product was dissolved in 50 μL nuclease-free water. The purified dsRNAs were quantified by spectroscopy. To deliver dsRNA into BPH, nymphs were reared on an artificial diet amended with dsRNA^27^, with some modifications to the rearing protocol. Previous results indicated that dsRNA feeding led to rapid and significant reduction in expression levels of BPH genes^28^. We used glass cylinders (15.0 × 2.5 cm diameter) as feeding chambers, with four dsRNA concentrations, 0.125, 0.075, 0.05, and 0.025 μg/μL. The dsRNA solution (final concentration, 0.05 μg/μL diet (determined from the concentrations just mentioned) was added to the artificial diet (20 μL), held between two layers of stretched Parafilm M membrane enclosed at the two open ends of the chamber (the diet capsule). The diet capsule was replaced every second day. The cylinders were covered with a piece of black cotton cloth, but the two ends with the artificial diet were exposed to light. Insects fed on the diets by puncturing the inner Parafilm M membrane of the diet capsule. Experimental insects were transferred into chambers and maintained on artificial diets for one day before initiation of the assays. Twenty 3^rd^ instar individuals were transferred into each chamber, and three chambers were used to create three independent biological replicates. The rearing experiments were carried out in a humidified growth cabinet at 26 ± 2 °C, 90% RH and a 16L:8D photoperiod. Mortality was recorded every other day.

### Influence of dietary dsRNA on biological performance parameters

We determined the effects of dsNlSPATA5 treatments on selected biological performance parameters. In preliminary experiments, we exposed second instar nymphs to the dsRNA construct, which led to over 95% mortality. Thereafter, we transferred 3^rd^ instar nymphs to capsules containing dsRNA-laced diet. At their fifth (final) instar (8 days), they were collected and nymphs were individually transferred into a glass jar (12 cm high × 10 cm) and reared on tillering rice plants under 26 ± 2 °C and 16L:8D. Eighty adult females and 80 adult males were collected separately at days 2 after emergence, and fresh body weight, soluble ovary protein content and soluble fat body protein content, soluble MAGs protein content and *NlSPATA5* expression level were determined. We paired individual newly-emerged females with a male. Each pair (♂ × ♀) was maintained in a glass jar (diameter 10 cm, height 12 cm) with rice seedlings under our standard conditions for oviposition. Eighteen copulating pairs were maintained to record duration of the pre-oviposition period, oviposition period, adult longevity, and fecundity for each pair. Rice stems were replaced daily during the pre-oviposition period, at two day intervals during the oviposition period and three day intervals during the female longevity period until the females died. The numbers of eggs laid on each rice stem was recorded under a light microscope. Eggs were scraped from the leaf sheaths and blades using a pin. Fecundity of 18 mated pairs was recorded as the average number of eggs laid.

### Protein Extraction and determinations

Protein was extracted from fat bodies and ovaries using a method similar to Gong *et al*.^29^. Individual adult females in all 15 females were dissected under a zoom stereomicroscope (model XTL20, Beijing Tech Instrument Co., Ltd., Beijing, China) in a cooled petri dish. Ovaries and fat bodies were removed and placed in separate, pre-weighed, ice-cold centrifuge tubes and then re-weighed using a Mettler-Toledo electronic balance (EC100 model; 1/10,000 *g* sensitivity). A proportional amount of NaCl solution (0.4 M NaCl: 1 M PMSF, v:v at a ratio of 20 mL NaCl solution to 1 g ovary or fat body) was added to the tube, homogenized on ice, and centrifuged at 16,000 × *g* at 4 °C for 20 min. The supernatant was collected after filtering the upper fat layer with glass fibers, placed at 4 °C overnight after adding ddH_2_O (1 supernatant: 10 ddH_2_O, v/v), and centrifuged again at 4,000 × *g* at 4 °C for 20 min. The protein sediment was dissolved with 1.5 ml pre-chilled 0.4 M NaCl solution after removing the supernatant. Protein from MAGs was extracted using the methods of Ge *et al*.^30^ Individual adult males in all 30 males were dissected under a zoom-stereomicroscope (model XTL20, Beijing Tech Instrument Co., Ltd., Beijing, China) in a cooled petri dish. MAGs were removed and placed in separate, pre-weighed, ice-cold Eppendorf tubes. To each tube, 600 μL of mixed solution (methanol/distilled water/acetic acid/methyl thioethanol; 80:18:2:0.1; v:v:v:v) was added. The contents were then homogenized on ice and centrifuged at 12,000 *×* *g* at 4 °C for 10 min. The supernatant was collected after removing the upper fat layer. Four hundred μL of mixed solution was added to the sediment in the tube, which was then centrifuged again and the supernatant collected.

We followed Bradford^31^ to measure protein content using Coomassie Brilliant Blue R250 (Shanghai Chemical Agent Co., Ltd., Shanghai, China). A standard curve was established based on a BSA standard protein (Shanghai Biochemistry Research Institute, Shanghai, China). The absorbance at 595 nm was determined in a UV755 B spectrometer (Shanghai Precision Instrument Co., Ltd., Shanghai, China). The protein content in the sample solution was calculated according to the standard curve. Protein determinations were repeated four times, with four independent biological samples.

### Observation of the number of yeast-like symbionts (YLS) in fat bodies

The procedure described in Noda^32^ was followed to measure the number of yeast-like symbionts (YLS) of fat bodies with blood cell counter (0.01 mm, 1/400 mm^2^) (25 * 16 model, Shanghai Qiujing Biochemical Reagent Co., Shanghai, China). We transferred 3^rd^ instar nymphs to capsules containing dsRNA-laced diet; at their fifth (final) instar (8 days), nymphs were individually transferred into a glass jar (12 cm high × 10 cm) and reared on tillering rice plants under 26 ± 2 °C and 16L:8D. We paired individual newly-emerged females with a male. Each pair (♂ × ♀) was maintained in a glass jar (diameter 10 cm, height 12 cm) with rice seedlings under our standard conditions for observation of the number of yeast-like symbionts (YLS) in fat bodies. Sixty uniform adult females were collected separately at 2 or 3 days after mating. Fat body was dissected from six adult females in treated or untreated control group and homogenized gently in 200 μl saline solution (0.9% NaCl). Two μL of homogenate were added to a hemocytometer (0.01 mm, 1/400 mm^2^) and the numbers of YLS were counted under microscope using a 5 point sampling method. The numbers of yeast-like symbionts were counted from 80 squares (unit, mm^2^) each time. Each treatment and control was replicated five times.

### Body weights

Five females or five males were used as a replicate at day 2. The insects were placed in pre-weighed centrifuge tubes and then weighed using a Mettler-Toledo electronic balance (EC100 model; 1/10,000 *g* sensitivity). Each treatment and each control experiment was replicated five times with five independent sets of insects.

### qPCR analysis

We isolated total RNA from the five newly-emerged females, using a SV Total Isolation System Kit (model Z3100, Promega Corporation, Madison, WI, USA). First-strand cDNA was synthesized in a 10 μL reaction volume containing 0.5 μg of RNA, 0.5 μL of PrimeScript RT enzyme mix I, 0.5 μL of Oligo dT primer (50 μM), 2 μL of random hexamers (100 μM), 2 μL 5× PrimeScript Buffer (for real time-PCR) and RNase–free dH_2_O up to a final volume of 10 μL, following the PrimeScript RT Kit instructions (TaKaRa Biotechnology, Dalian, China). The cDNA was reverse transcribed using the following program: 37 °C for 15 min, 85 °C for 5 s and 4 °C for 5 min.

We similarly isolated total RNA from the dsRNA-treated and control adults. Portions (2 μL of the synthesized first-strand cDNA were amplified by qPCR in 20 μL reaction mixtures using a CFX96 real-time PCR system (Bio-Rad Co. Ltd., California, USA). We used two qPCR programs. For *NlSPATA5*, 94 °C for 2 min, followed by 40 cycles of 94 °C for 5 s, 56 °C for 30 s and 72 °C for 30 s. For *Nlvitellogen (Nlvg*), 94 °C for 2 min, followed by 40 cycles of 94 °C for 5 s, 59.7 °C for 30 s and 72 °C for 30 s. *NlSPATA5* (NLU004890) and *Nlvg* (AB353856) mRNA levels were separately quantified in relation to the stable expression^19^ of constitutive *actin-1* (EU179846). Primers used for qPCR analysis are listed in Table 1. After amplification, a melting curve analysis was performed in triplicate and the results were averaged. The values were calculated using three independent biological samples and the 2^−△△CT^ method^33^ was used for the analysis of relative *NlSPATA5* expression level.

### Statistical analysis

Before performing an analysis of variance (ANOVA), data were evaluated for normality and homogeneity of variance using a Bartlett test. Based on these evaluations, no transformations were needed. The results presented in figures are expressed as the means ± S.E. Two-way (days post-emergence and dsRNA treatment) ANOVAs were performed to analyze data in Fig. 1B. One-way ANOVAs were performed to analyze all other data except for Fig. 1B. Multiple comparisons of the means were conducted using Fisher’s Protected Significant Difference (PLSD) test. All analysis were conducted using the data processing system (DPS) of Tang and Feng^34^.

## Additional Information

**How to cite this article**: Ge, L.-Q. *et al*. Suppressing male spermatogenesis-associated protein 5-like gene expression reduces vitellogenin gene expression and fecundity in *Nilaparvata lugens* Stål. *Sci. Rep.* **6**, 28111; doi: 10.1038/srep28111 (2016).

## Figures and Tables

**Table 1:** PCR primers used in this study.

**Primers**	**Primer sequence**	**Product size**
For real-time PCR		
Q-*NlSPATA5*-F	CAGGTCAAGGACGATACGG (Tm = 59.7 °C)	127 bp
Q-*NlSPATA5*-R	CCTTCACTTTCAGCGATT (Tm = 52.7 °C)	
Q-*Nlvg*-F	GTGGCTCGTTCAAGGTTATGG (Tm = 58.0 °C)	200 bp
Q-*Nlvg*-R	GCAATCTCTGGGTGCTGTTG (Tm = 60.2 °C)	
Actin-F	TGCGTGACATCAAGGAGAAGC (Tm = 60.0 °C)	186 bp
Actin-R	CCATACCCAAGAAGGAAGGCT (Tm = 60.0 °C)	
For dsRNA synthesis		
* NlSPATA5*-F	TAATACGACTCACTATAGGG (T7 promoter) CGCTGAAAGTGAAGGA (Tm = 51.6 °C)	407 bp
* NlSPATA5*-R	TAATACGACTCACTATAGGG (T7 promoter) GTCTGCAAGCAGGATT (Tm = 51.6 °C)	
For dsRNA synthesis		
* NlGFP*-F	TAATACGACTCACTATAGGG (T7promoter) AAGGGCGAGGAGCTGTTCACCG-3 (Tm = 60 °C)	688 bp
* NlGFP*-R	TAATACGACTCACTATAGGG (T7protomer) CAGCAGGACCATGTGATCGCGC-3′ (Tm = 56 °C)	

## References

[b1] Heinrichs E. A. In Planthoppers: Their Ecology and Management. (eds Denno R. F., Perfect T. J.) 571–614 (Chapman and Hall Press, 1994).

[b2] Chelliah S. & Heinrichs E. A. Factors affecting insecticide-induced resurgence of the brown planthopper, *Nilaparvata lugens* on rice. Environ. Entomol. 9, 773–777 (1980).

[b3] Wang L. P., She J., Ge L. Q. & Wu J. C. Insecticide-induced increase in the protein content of male accessory glands and its effect on the fecundity in the brown phanthopper *Nilaparvata lugens* Stål (Hemiptera: Delphacidae). Crop. Prot. 29, 1280–1285 (2010).

[b4] Ge L. Q., Cheng Y., Wu J. C. & Jahn G. C. Proteomic analysis of insecticide triazophos-induced mating-responsive proteins of *Nilaparvata lugens* Stål (Hemiptera: Delphacidae). J. Proteome Res. 10, 4597–4612 (2011). 21800909 10.1021/pr200414g

[b5] Gillott C. Male accessory gland secretions: modulators of female reproductive physiology and behavior. Annu. Rev. Entomol. 48, 163–184 (2003). 12208817 10.1146/annurev.ento.48.091801.112657

[b6] Jin Z. Y. & Gong H. Male accessory gland derived factors can stimulate oogenesis and enhance oviposition in *Helicoverpa armigera* (Lepidoptera: Noctuidae). Arch. Insect Biochem. Physiol. 46, 175–185 (2001). 11304751 10.1002/arch.1027

[b7] Himuro C. & Fujisake K. Males of the seed bug *Togo hemipterus* (Heteroptera: Lygaeidae) use accessory gland substances to inhibit remating by females. J. Insect Physiol. 54, 1538–1542 (2008). 18835395 10.1016/j.jinsphys.2008.09.002

[b8] Avila F. W., Sirot L. K., LaFlamme B. A., Rubinstein C. D. & Wolfner M. F. Insect seminal fluid proteins: identification and function. Annu. Rev. Entomol. 56, 21–40 (2011). 20868282 10.1146/annurev-ento-120709-144823 PMC3925971

[b9] Klowden M. J. Sexual receptivity in *Anopheles gambiae* mosquitoes: absence of control by male accessory gland substances. J. Insect Physiol. 47, 661–666 (2001). 11356412 10.1016/s0022-1910(00)00127-x

[b10] Ram K. R. & Wolfner M. F. A network of interactions among seminal proteins underlies the long-term postmating response in *Drosophila*. Proc. Natl. Acad. Sci. USA 106, 15384–15389 (2009). 19706411 10.1073/pnas.0902923106 PMC2741260

[b11] Maran C., Tassone E., Masola V. & Onisto M. The story of SPATA2 (Spermatogenesis-Associated Protein 2): From Sertoli Cells to Pancreatic Beta-Cells. Curr Genom 10, 361–363 (2009). 10.2174/138920209788920976 PMC2730000 20119533

[b12] Huo S., Du W., Shi P., Si Y. & Zhao S. The role of spermatogenesis-associated protein 6 in testicular germ cell tumors. Int J clin Exp Pathol 8, 9119–9125 (2015). 26464655 PMC4583887

[b13] Bonilla E. & Xu E. Y. Identification and characterization of novel mammalian spermatogenic genes conserved from fly to human. Bas Sci Reproduct Med 14, 137–142 (2008). 10.1093/molehr/gan002 18256174

[b14] Snider J., Thibault G. & Houry W. A. The AAA+ superfamily of functionally diverse proteins. Genome Biol 9, 216 (2008). 18466635 10.1186/gb-2008-9-4-216 PMC2643927

[b15] Liu Y., Black J., Kisiel N. & Kulesz-Martin M. F. SPAF, a new AAA-protein specific to early spermatogenesis and malignant conversion. Oncogene 19, 1579–1588 (2000). 10734318 10.1038/sj.onc.1203442

[b16] Forstbauer L. M. . Genome-wide pooling approach identifies SPATA5 as a new susceptibility locus for alopecia areata. Eur J. Hum. Genet. 20, 326–332 (2012). 22027810 10.1038/ejhg.2011.185 PMC3283178

[b17] Tanaka A. J. . Mutations in *SPATA5* are associated with microcephaly, intellectual disability, seizures, and hearing loss. Am. J. Hum. Genet. 97, 457–464 (2015). 26299366 10.1016/j.ajhg.2015.07.014 PMC4564988

[b18] Ge L. Q., Wu J. C., Zhao K. F., Chen Y. & Yang G. Q. Induction of Nlvg and suppression of *Nljhe* gene expression in *Nilaparva*ta *lugens* (Stål) (Hemiptera: Delphacidae) adult females and males exposed to two insecticides. Pestic. Biochem. Physiol. 98, 269–278 (2010a).

[b19] Ge L. Q. . Silencing a sugar transporter gene reduces growth and fecundity in the brown planthopper, *Nilaparvata lugens* (Stål) (Hemiptera: Delphacidae). Sci. Reps. 5, 12194 (2015). 10.1038/srep12194 PMC4505327 26185058

[b20] Li L. . Jinggangmycin increases fecundity of the brown planthopper, *Nilaparvata lugens* (Stål) via fatty acid synthase gene expression. J. Proteomics 130, 140–149 (2015). 26388431 10.1016/j.jprot.2015.09.022

[b21] Sassaki T., Kawamura M. & Ishikawa H. Nitrogen recycling in brown planthooper *Nilaparvata lugens*: Involvement of yeast-like endosymbionts in uric acid metabolism. J. Insect Physiol. 42, 125–129 (1996).

[b22] Wang G. C. . Relationship between yeast-like symbiotes and amino acid requirements in the rice brown planthopper, *Nilaparvata lugens* Stål (Homoptera: Delphacidae). Acta Entomol. Sin. 48, 483–490 (2005).

[b23] Wilkinson T. L. & Ishikawa H. On the functional significance of symbiotic microogranisms in the Homoptera: a comparative study of *Acyrthosiphon pisum* and *Nilaparvata lugens*. Physiol. Entomol. 26, 86–93 (2001).

[b24] Lee Y. H. & Hou R. F. Physiological roles of a yeast-like symbiote in reproduction and embryonic development of the brown planthopper. J. Insect Physiol. 33, 852–860 (1987).

[b25] Thornhill R. & Alcock J. The Evolution of Insect Mating Systems. (Harvard University Press, 1983).

[b26] Stanley D. & Kim Y. Eicosanoid signaling in insects: from discovery to plant protection. Crit. Rev. Plant Sci. 33, 20–63 (2014).

[b27] Fu Q., Zhang Z. T., Lai F. X. & Hu C. A chemically defined diet enables the continuous rearing of the brown planthopper, *Nilaparvata lugens* (Stål). Appl. Entomol. Zool. 36, 111–1116 (2001).

[b28] Chen J. . Feeding-based RNA interference of a trehalose phosphate synthase gene in the brown planthopper, Nilaparavata lugens. Insect Mol. Biol. 19, 777–786 (2010). 20726907 10.1111/j.1365-2583.2010.01038.x

[b29] Gong H., Zhai C. H., Wei D. Y. & Zhang J. Z. On the vitellogenesis of *Coccinella septempunctata* L: the occurrence of vitellogenin as influenced by artificial diet. Acta. Entomol. Sin. 23, 252–257 (1980).

[b30] Ge L. Q., Wang L. P., Zhao K. F., Wu J. C. & Huang L. J. Mating pair combinations of insecticide-treated male and female *Nilaparvata lugens* Stål(Hemiptera: Delphacidae) planthoppers influence protein content in the male accessory glands (MAGs) and vitellin content in both fat bodies and ovaries of adult females. Pestic. Biochem. Physiol. 98, 279–288 (2010b).

[b31] Bradford M. M. A rapid and sensitive method for the quantitation of microgram quantities of protein utilizing the principle of protein-dye binding. Anal. Biochem. 72, 248–254 (1976). 942051 10.1016/0003-2697(76)90527-3

[b32] Noda H. Preliminary histological observation and population dynamics of intracellular yeast-like symbiotes in the smaller brown planthopper, *Laodelphax striatellus* (Homoptera: Delphacidae). Appl. Entomol. Zool. 9, 275–277 (1974).

[b33] Livak K. J. & Schmittgen T. D. Analysis of relative gene expression data using real-time quantitative PCR and the 2^−△△CT^ method. Methods 25, 402–408 (2001). 11846609 10.1006/meth.2001.1262

[b34] Tang Q. & Feng M. G. In DPS Data Processing System for Practical Statistics-4. (eds Tang Q. & Feng M. G.) 47–71 (Scientific Press, 2002).

